# Using Population Health Measures to Evaluate the Environmental Burden of Cancer at the County Level

**DOI:** 10.5888/pcd16.180530

**Published:** 2019-04-11

**Authors:** Lia C. Scott, Lawrence E. Barker, Lisa C. Richardson

**Affiliations:** 1 Centers for Disease Control and Prevention, National Center for Chronic Disease Prevention and Health Promotion, Atlanta, Georgia

## Abstract

**Introduction:**

Burden of disease is often defined by using epidemiologic measures. However, there may be latent aspects of disease burden that are not factored into these types of estimates. This study quantified environmental burden of disease by using population health indicators and exploratory factor analysis at the county level across the United States.

**Methods:**

Ninety-nine variables drawn from public use data sets from 2010 to 2016 were used to create a multifactor index — the burden index. We applied principal components analysis with promax rotation to allow the factors to correlate. Correlation coefficients for each factor and the outcome of interest, age-adjusted cancer death rate, were calculated. We used both unadjusted and adjusted linear regression techniques.

**Results:**

The final additive county-level index included 9 factors that explained 68.3% of the variance in the counties and county equivalents. The burden index had a moderate association with the age-adjusted cancer death rates (*r* =.48, *P* <.001), and adjusted linear regression with all 9 factors explained 34% of the variance in the age-adjusted cancer death rate. Results were mapped, and the geographic distribution of both the burden index and age-adjusted cancer mortality were assessed. There are distinct geospatial patterns for both.

**Conclusions:**

Results from this study show potential areas of need, as well as the importance of including environmental variables in the study of cancer etiology. Future studies can aim to validate these findings by quantifying burden as it relates to overall cancer mortality by using epidemiologic measures, along with other confirmatory statistical methods.

**Summary**

**What is already known about this topic?**

There is a burgeoning interest in the collective health of communities and the social determinants of health that influence those outcomes. These determinants and their relationship to cancer etiology and outcomes are of public health concern, as research continues to embrace a multilevel framework for understanding the cancer continuum.

**What is added by this report?**

A single index at the county level was created that quantifies environmental burden using population health indicators. It highlights the index’s relationship to cancer mortality and demonstrates the geographic distribution of the index.

**What are the implications for public health practice?**

The environment burden index can enhance public health practice and assist in program planning and cancer control efforts.

## Introduction

Epidemiologic and economic measures, such as the mortality, morbidity, or financial burden of disease, can define its total burden (1). Studies examining burden of disease often use epidemiologic measures, quality-adjusted life-years, or disability-adjusted life-years (2,3). However, the disease burden might not solely depend on these measures. Less tangible, or latent, aspects of disease burden may exist (4–6). This complex network of factors, both proximal and distal, can influence individual health outcomes (7,8).

Interest in measuring population health outcomes and ranking communities (eg, the Social Vulnerability Index [9]) has increased (10–12). Much literature focuses on environmental hazards or disasters. Few studies have considered reducing the dimensionality of the factors that could influence cancer. We considered disease burden as a latent variable: an unobservable factor only reachable through data reduction (13). Because we defined burden as an agglomeration of community indicators at the county level that exacerbate poor health outcomes, we addressed it through statistical methods.

## Methods

### Data

To quantify and examine burden, we collected socioeconomic, demographic, and other contextual data from 2010 through 2016 for all US counties and county equivalents. Texas has the highest number of counties (n = 254) followed by Georgia (n = 159). The scope of our analysis also included US territories. All statistical analyses were conducted in SAS 9.4 (SAS Institute Inc), with mapping conducted in ArcGIS 10.5 (Esri Corp). Data were combined from multiple public-use sources. Over 300 variables were collected and combined; after removing redundancies and testing for multicollinearity, 99 were retained for analysis, and 56 were included in the final output (Table 1).

**Table 1:** Variables in Final Calculated Index of Environmental Burden of Cancer, County and County Equivalents, 2010–2016^a^

Variable Name^b^	Description	Source
AsianNonHispanicPct2010	Percentage Non-Hispanic Asian, 2010	US Department of Agriculture^c^
f1461311	Median home value 2011–2015	Area Health Resource Files^d^
Iasian10	Isolation Index(Asian): Minority-weighted average across census tracts within a county that reflects the probability of contact among members of Asian racial group (derived from Census estimate).	Spatial Impact Factor Database^e^
f1461411	Median gross rent 2011–2015	Area Health Resource Files^d^
PerCapitaInc	Per capita income in the past 12 months), 2010–2014 (in 2014 inflation adjusted dollars)	US Department of Agriculture^c^
ForeignBornEuropePct	Percentage of persons born in Europe, 2010–2014	US Department of Agriculture^c^
MedHHInc2014	Median household income, 2014 (in 2014 dollars)	US Department of Agriculture^c^
PctEmpServices	Percentage employed in services, 2010–2014	US Department of Agriculture^c^
MDPC_15	Total medical doctors, not employed by the federal government, per 100,000 population, 2015	Area Health Resource Files^d^
PCPPC_15	Total office-based and hospital-based primary care (general family medicine, general practice, general internal medicine and general pediatrics) physicians, not employed by the federal government, per 100,000 population, 2015	Area Health Resource Files^d^
PCT_OBESE_ADULTS13	Adult obesity rate, 2013	US Department of Agriculture^f^
CurrSmk0810	Percentage of persons aged ≥18 who reported smoking at least 100 cigarettes in their life, and now smoking cigarettes some days or every day at the time of interview	National Cancer Institute/Behavioral Risk Factor Surveillance System^g^
Ed2HSDiplomaOnlyPct	Percentage of persons with a high school diploma or GED only, adults 25 and over, 2010–2014	US Department of Agriculture^c^
BlackNonHispanicPct2010	Percentage of non-Hispanic African Americans, 2010	US Department of Agriculture^c^
pvBR59	Percentage of total population for whom poverty data exist who are black and rural	Spatial Impact Factor Database^e^
Iblack10	Isolation Index(Black): Minority-weighted average across census tracts within a county that reflects the probability of contact among members of Black racial group (derived from Census estimate).	Spatial Impact Factor Database^e^
FemaleHHPct	Percentage of female-headed family households of total households, 2010–2014	US Department of Agriculture^c^
PFSR_14	Percentage Food Stamp/SNAP recipients, 2014	Area Health Resource Files^d^
PCT_FREE_LUNCH14	Percentage of students eligible for free lunch, 2014	US Department of Agriculture^f^
PovertyAllAgesPct2014	Percentage of people with income below the federal poverty level, 2014	US Department of Agriculture^c^
UnempRate2015	Unemployment rate, 2015	US Department of Agriculture^c^
PHHSSI	Percentage households with Supplemental Security Income (SSI), 2011–2015	Area Health Resource Files^d^
SNAPSPTH16	SNAP-authorized stores per 1,000 population, 2016	US Department of Agriculture^f^
PCT_LACCESS_HHNV15	Percentage of households with no car and low access to grocery store, 2015	US Department of Agriculture^f^
PCT_DIABETES_ADULTS13	Adult diabetes rate, 2013	US Department of Agriculture^f^
WhiteNonHispanicPct2010	Percentage non-Hispanic white, 2010	US Department of Agriculture^c^
Iwhite10	Isolation Index(White): Minority-weighted average across census tracts within a county that reflects the probability of contact among members of White racial group (derived from Census estimate).	Spatial Impact Factor Database
HispanicPct2010	Percentage Hispanic people, 2010	US Department of Agriculture^c^
ForeignBornMexPct	Percentage of people born in Mexico, 2010–2014	US Department of Agriculture^c^
IHisp10	Isolation Index(Hispanic): Minority-weighted average across census tracts within a county that reflects the probability of contact among members of Hispanic ethnic group (derived from Census estimate).	Spatial Impact Factor Database
ForeignBornCaribPct	Number of people born in the Caribbean, 2010–2014	US Department of Agriculture^c^
ForeignBornPct	Percentage of total population foreign born, 2010–2014	US Department of Agriculture^c^
pvHR59	Percentage of total population for whom poverty data exists who are Hispanic and rural	Spatial Impact Factor Database^e^
pvHU59	Percentage of total population for whom poverty data exists who are Hispanic and urban	Spatial Impact Factor Database^e^
PCT_WIC15	Percentage of population receiving WIC benefits, 2015	US Department of Agriculture^c^
FOODINSEC_CHILD_03_11	Child food insecurity (percentage of households, multiple-year average), 2003–2011	US Department of Agriculture^f^
PCT_SBP15	Percentage of population participating in School Breakfast Program, 2015	US Department of Agriculture^f^
PCT_NSLP15	Percentage of population participating in National School Lunch Program, 2015	US Department of Agriculture^f^
PC_FFRSALES12	Expenditures per capita on fast food, 2012	US Department of Agriculture^f^
FOODINSEC_13_15	Percentage of population experiencing household food insecurity (3-year average), 2013–2015	US Department of Agriculture^f^
FQHCDENS_16	Number of Federally Qualified Health Centers per square mile, 2016	Area Health Resource Files^d^
PopDensity2010	Population density, 2010	US Department of Agriculture^c^
HOSPDENS_14	Total number hospitals per square mile, 2014	Area Health Resource Files^d^
f1348310	Median age, 2010	Area Health Resource Files^d^
HH65PlusAlonePct	Percentage of persons ≥65 living alone, 2010–2014	US Department of Agriculture^c^
PHHSocSI	Percentage of households with Social Security income 2011–2015	Area Health Resource Files
xwood10	Percentage of households using wood for heat	Spatial Impact Factor Database^e^
noplum10	Percentage of households lacking complete plumbing	Spatial Impact Factor Database^e^
xvac10	Proportion of all housing units that were vacant, 2010	Spatial Impact Factor Database^e^
PW90Work	Percentage workers aged ≥16 living ≥90 min from work, 2011–2015	Area Health Resource Files^d^
PW6089Work	Percentage workers aged ≥16 living 60–89 min from work, 2011–2015	Area Health Resource Files^d^
f1458511	Percentage employed in construction 2011–2015	Area Health Resource Files^d^
PctEmpGovt	Percentage employed in government, 2010–2014	US Department of Agriculture^c^
PC_FSRSALES12	Expenditures per capita, restaurants, 2012	US Department of Agriculture^f^
f1458811	Percentage workers in other industries, 2011–2015^h^	Area Health Resource Files^d^
f1458711	Percentage employed in manufacturing, 2011–2015	Area Health Resource Files^d^

Abbreviations: GED, general equivalency diploma; SNAP, Supplemental Nutrition Assistance Program; WIC, Special Supplemental Nutrition Program for Women, Infants, and Children.

a The burden index is a combination of community indicators at the county level that exacerbate poor health outcomes, defined statistically through Principal Components Analysis of 99 variables reduced to 56 variables in 9 factors. The factors are combined in an additive county-level index that explained 68.3% of the variance in the counties and county equivalents.

b Variable names are derived from their original sources.

c Atlas of Rural and Small-Town America (30).

d Area Health Resource Files (31).

e Mobley, LR. (32).

f Food Environment Atlas (33).

g Model-Based Small Area Estimates of Cancer Risk Factors & Screening Behaviors (34).

h Other Industries include wholesale trade; retail trade; transportation and warehousing, and utilities; information; finance and insurance, and real estate and rental and leasing; professional, scientific, and management, and administration and waste management services; arts, entertainment, and recreation, and accommodation and food services; other services, except public administration; and public administration.

Variables were selected via literature that identified key population health indicators. These variables included, but were not limited to, poverty, income, food access, segregation, social capital, and demographic characteristics (7,8). All data were merged on the State-County FIPS (federal information processing standard) code. Following the Social Vulnerability Index methods (9), count variables were normalized as either per-capita values, percentages, or density functions. Missing data were handled by using unweighted hot deck imputation (14). This method, used by the US Census Bureau, reduces nonresponse bias (15), is nonparametric, and is therefore insensitive to model misspecification. Approximately 80% of the observations had complete data, and the remaining variables had no more than 4.3% missing. Variables were then standardized by using *z* score standardization.

The outcome of interest (dependent variable) was overall county-level, age-adjusted cancer death rate from 2010 through 2014 from US cancer statistics. These data were obtained from the National Vital Statistics System public use data file (16).

### Statistical methods

Factor analysis is a method for reducing a large number of variables to a smaller set of grouped variables (factors). Factors minimize information loss by reducing observed variances into measures of latent constructs (17). We had no a priori theory of the latent constructs, so we conducted an exploratory factor analysis (17).

We used principal components analysis, a commonly used method of exploratory factor analysis. We used a varimax then promax (oblique) rotation, which allows the factors to have non-zero correlations, and used Kaiser criterion with 100 iterations for component selection (18,19). Factor analysis was deemed suitable after review of variable correlations, the Kaiser Measure of Sampling Adequacy (Kaiser MSA), and variable communalities (17). Factors were extracted, cardinality was determined on the basis of correlation coefficients with the outcome, an additive burden index was computed for each county, and each factor was weighted equally. Negative factors were expected to decrease burden, whereas positive factors were expected to increase burden.

Loadings with an absolute value of 0.5 or higher were flagged in the factor analysis. Twenty-one factors were extracted via the Kaiser criterion. Factors that had fewer than or equal to 2 variables loading were not retained because of underidentification. The factors were regressed on the outcome of interest: overall county-level, age-adjusted cancer death rate from 2010 through 2014, in both unadjusted and adjusted analyses. The burden index was also regressed on the outcome. The results from the burden index were mapped across the United States, along with overall cancer death rate.

This work was a secondary analysis of publicly available data; institutional review board review was not required.

## Results

All variables had a correlation of at least 0.3 with at least one other variable. The Kaiser MSA was 0.86. The communalities were all above 0.4, confirming that each variable shared some communality with other variables.

Our analysis yielded 21 factors of which 9 were retained to create the composite index of underlying population health indicators. Factors that were retained had 3 or more variables load on them with a minimum value of 0.5. These factors, described below, explained 68.3% of the variance among counties and county equivalents. Factor names were based on the highest loading variables and should not be interpreted beyond being representative of a latent construct (Table 2). Regression results appear in Table 3.

**Table 2:** Factor Loadings^a^ and Communalities Based on a Principal Components Analysis With Promax Rotation Resulting in 56 Items (N = 3,233), Index of Environmental Burden of Cancer, County and County Equivalents, 2010–2016^b^

Variable Name	Asian Race and Isolation; High Income and Home Value	Black Race and Isolation;	Hispanic Ethnicity and Isolation	Food Insecurity	Hospital and Population Density	Older Age	Housing	Stress (Long Commute)	Employed in Government or Other Services	Communality
AsianNonHispanicPct2010	1.01	—c	—c	—c	—c	—c	—c	—c	—c	0.82
f1461311	0.91	—c	—c	—c	—c	—c	—c	—c	—c	0.87
Iasian10	0.91	—c	—c	—c	—c	—c	—c	—c	—c	0.80
f1461411	0.83	—c	—c	—c	—c	—c	—c	—c	—c	0.88
PerCapitaInc	0.77	—c	—c	—c	—c	—c	—c	—c	—c	0.88
ForeignBornEuropePct	0.76	—c	—c	—c	—c	—c	—c	—c	—c	0.75
MedHHInc2014	0.64	—c	—c	—c	—c	—c	—c	—c	—c	0.91
PctEmpServices	0.64	—c	—c	—c	—c	—c	—c	—c	—c	0.83
MDPC_15	0.62	—c	—c	—c	—c	—c	—c	—c	—c	0.71
PCPPC_15	0.61	—c	—c	—c	—c	—c	—c	—c	—c	0.69
PCT_OBESE_ADULTS13	−0.54	—c	—c	—c	—c	—c	—c	—c	—c	0.76
CurrSmk0810	−0.56	—c	—c	—c	—c	—c	—c	—c	—c	0.75
Ed2HSDiplomaOnlyPct	−0.66	—c	—c	—c	—c	—c	—c	—c	—c	0.76
BlackNonHispanicPct2010	—c	0.98	—c	—c	—c	—c	—c	—c	—c	0.96
pvBR59	—c	0.94	—c	—c	—c	—c	—c	—c	—c	0.85
Iblack10	—c	0.87	—c	—c	—c	—c	—c	—c	—c	0.92
FemaleHHPct	—c	0.82	—c	—c	—c	—c	—c	—c	—c	0.87
PFSR_14	—c	0.78	—c	—c	—c	—c	—c	—c	—c	0.87
PCT_FREE_LUNCH14	—c	0.78	—c	—c	—c	—c	—c	—c	—c	0.80
PovertyAllAgesPct2014	—c	0.72	—c	—c	—c	—c	—c	—c	—c	0.91
UnempRate2015	—c	0.69	—c	—c	—c	—c	—c	—c	—c	0.66
PHHSSI	—c	0.62	—c	—c	—c	—c	—c	—c	—c	0.74
SNAPSPTH16	—c	0.59	—c	—c	—c	—c	—c	—c	—c	0.65
PCT_LACCESS_HHNV15	—c	0.58	—c	—c	—c	—c	—c	—c	—c	0.72
PCT_DIABETES_ADULTS13	—c	0.58	—c	—c	—c	—c	—c	—c	—c	0.81
WhiteNonHispanicPct2010	—c	−0.68	—c	—c	—c	—c	—c	—c	—c	0.96
Iwhite10	—c	−0.69	—c	—c	—c	—c	—c	—c	—c	0.93
HispanicPct2010	—c	—c	0.99	—c	—c	—c	—c	—c	—c	0.93
ForeignBornMexPct	—c	—c	0.98	—c	—c	—c	—c	—c	—c	0.87
IHisp10	—c	—c	0.97	—c	—c	—c	—c	—c	—c	0.91
ForeignBornCaribPct	—c	—c	0.97	—c	—c	—c	—c	—c	—c	0.89
ForeignBornPct	0.50	—c	0.77	—c	—c	—c	—c	—c	—c	0.92
pvHR59	—c	—c	0.77	—c	—c	—c	—c	—c	—c	0.71
pvHU59	—c	—c	0.71	—c	—c	—c	—c	—c	—c	0.61
PCT_WIC15	—c	—c	—c	0.89	—c	—c	—c	—c	—c	0.82
FOODINSEC_CHILD_03_11	—c	—c	—c	0.85	—c	—c	—c	—c	—c	0.83
PCT_SBP15	—c	—c	—c	0.85	—c	—c	—c	—c	—c	0.85
PCT_NSLP15	—c	—c	—c	0.71	—c	—c	—c	—c	—c	0.76
PC_FFRSALES12	—c	—c	—c	0.57	—c	—c	—c	—c	—c	0.72
FOODINSEC_13_15	—c	—c	—c	0.53	—c	—c	—c	—c	—c	0.45
FQHCDENS_16	—c	—c	—c	—c	1.00	—c	—c	—c	—c	0.91
PopDensity2010	—c	—c	—c	—c	0.97	—c	—c	—c	—c	0.92
HOSPDENS_14	—c	—c	—c	—c	0.90	—c	—c	—c	—c	0.89
f1348310	—c	—c	—c	—c	—c	0.87	—c	—c	—c	0.90
HH65PlusAlonePct	—c	—c	—c	—c	—c	0.85	—c	—c	—c	0.80
PHHSocSI	—c	—c	—c	—c	—c	0.85	—c	—c	—c	0.86
xwood10	—c	—c	—c	—c	—c	—c	0.88	—c	—c	0.74
noplum10	—c	—c	—c	—c	—c	—c	0.57	—c	—c	0.48
xvac10	—c	—c	—c	—c	—c	—c	0.53	—c	—c	0.67
PW90Work	—c	—c	—c	—c	—c	—c	—c	0.81	—c	0.66
PW6089Work	—c	—c	—c	—c	—c	—c	—c	0.73	—c	0.68
f1458511	—c	—c	—c	—c	—c	—c	—c	0.52	—c	0.61
PctEmpGovt	—c	—c	—c	—c	—c	—c	—c	—c	0.72	0.60
PC_FSRSALES12	—c	—c	—c	—c	—c	—c	—c	—c	0.61	0.66
f1458811	0.56	—c	—c	—c	—c	—c	—c	—c	0.52	0.81
f1458711	—c	—c	—c	—c	—c	—c	—c	—c	−0.55	0.80

a Factor loadings can be interpreted as correlations between the variable and the factor the variable loads on. All variables presented in this table have a moderate to large associations with their corresponding factor. Factor loadings <|0.5| are suppressed.

b These 56 items are the variables listed in the first column of the table. These are the variables included in the output from the principal components analysis; 3,233 represents the number of counties included in the analysis. The Index of Environment Burden of Cancer is an additive index created from extracted county level values for each factor. The factors are the items in the first row of the table (ie, Asian race and isolation; high income and home value).

c Indicates factor loadings were <.5 and therefore suppressed.

**Table 3:** Regression Results for Factors and Index on Age-Adjusted Overall Cancer Mortality Rates, 2010–2014, Index of Environmental Burden of Cancer, County and County Equivalents

Factor Name	Bivariate Linear Regressions					Multivariate Linear Regression^a^		
	Intercept	β	SE	*P* Value	*r* ^2^	β	SE	*P* Value
Intercept	—	—	—	—	—	176.74	0.41	<.001
Asian race and isolation; high income & home value	176.39	−10.39	0.48	<.001	0.13	−7.1	0.53	<.001
Black race and isolation;	176.57	10.82	0.45	<.001	0.16	9.65	0.46	<.001
Hispanic ethnicity and isolation	176.59	7.19	0.48	<.001	0.07	−8.58	0.47	<.001
Food insecurity	176.57	6.34	0.48	<.001	0.05	2.02	0.47	<.001
Hospital and population density	176.75	0.36	0.61	.56	0.0001	2.03	0.54	.002
Older age (≥65 y)	176.75	3.8	0.49	<.001	0.02	0.06	0.50	.90
Housing	176.74	−0.12	0.5	0.81	0	−1.6	0.49	.001
Stress (long commute to work)	176.78	4.51	0.49	<.001	0.03	1.88	0.42	<.001
Employed in government	176.72	−1.84	0.5	.002	0.004	1.29	0.46	.053
Burden index	176.38	3.99	0.13	<.001	0.23	—	—	—

Abbreviations: —, variable not included in the model; SE, standard error.

a *r^2^* = 0.34.

**Asian race and isolation; high income and home value**, factor 1, identified Asian race, median home value, Asian residential isolation, median gross rent, and per-capita income along with 10 other variables. It explained 14.6% of the variation among US counties and county equivalents. The percentage of adults with obesity, current smokers, and the percentage of persons with a high school diploma or General Education Development only were the only variables that loaded negatively on the factor. This factor had a negative association with the overall cancer death rate (*r* = −0.36, *P* <.001).

**Black race and isolation, **factor 2, identified black race, the percentage of the population that is in poverty, black and rural, black residential isolation, and 11 other variables. This factor explained 12.5% of the variance among US counties and county equivalents. Only the percentage of non-Hispanic white population, and white isolation loaded negatively on this factor. This factor had a positive association with the overall cancer death rate (*r* = 0.39, *P* <.001).

**Hispanic ethnicity and isolation, **factor 3, identified Hispanic ethnicity, the percentage of persons born in Mexico and the Caribbean, Hispanic residential isolation, and 3 other variables. This variable is characterized mainly by the 4 aforementioned variables, which all have loadings greater than or equal to 0.97. This factor explained 9.8% of the variance among the counties and county equivalents and was negatively associated with the overall cancer death rate (*r* = −0.26, *P* <.001).

**Food insecurity,** factor 4, identified food insecurity. The highest loading variables were the percentage of Women, Infants, and Children (WIC) program participants, the percentage of households classified with child food insecurity, and the percentage of School Breakfast Program participants. This factor identified numerous variables that indicate safety nets for populations who are food insecure. This factor explained 7.98% of the variance among the counties and county equivalents. It was positively associated with the overall cancer death rate (*r* =.23, *P* <.001).

**Hospital and population density,** factor 6, identified hospital and population density by using 3 variables. Federally qualified health center density was the highest loading variable, followed by population density and total hospital density. This factor explained 4.97% of the variance and was not significantly associated with the overall cancer death rate (*r* = 0.01, *P* = .55).

**Older age,** factor 7, had only 3 variables load on it, and it identified persons aged 65 or older. It is characterized by median age, the percentage of persons aged 65 years or older and living alone, and the percentage of households with Social Security income. The factor explained 7.7% of the variance in the data set. The factor was positively associated with the overall cancer death rate (r = 0.14, *P* <.001).

**Housing,** factor 8, identified poor housing issues and explained 4.4% of the variance among counties and county equivalents. It was characterized by 3 variables; the percentage of households using wood for heat had the highest loading. The percentage of households lacking complete plumbing and the proportion of housing units that were vacant also loaded on this factor. The factor was not significantly associated with the overall cancer death rate (*r* = −0.01, *P* = .71).

**Stress (long commute),** factor 10, identified stress in terms of work and work commute and explained 3.4% of the variance. Three variables loaded on this factor, two representing commute to work, and one representing a high-stress work environment. It is characterized by the percentage of workers who travel at least one hour to work, and the percentage of workers in construction. The factor had a small positive association with the overall cancer death rate (*r* = 0.16, *P* < .001).

**Employed in government,** factor 13, the final factor in the equation, was identified by 4 variables and explained 2.96% of the variance among county and county equivalents. The highest loading variable was the percentage employed in government. This was followed by expenditures per capita in restaurants and the percentage of workers in other industries. These other industries included trade, transportation, information, and finance and professional, scientific, and management services. The percentage employed in manufacturing negatively loaded on this factor. The factor had a small negative association with the overall cancer death rate (*r* = −0.07, *P* < .001).

**Burden index.** The burden index was positively associated with the outcome. A 1-unit increase in the index corresponded to an approximately 4-unit increase in the overall cancer death rate, on average. The index explained 23% of the variance in overall cancer death rates. In adjusted regression analysis, all factors were significant predictors of the outcome except the older age factor. This model explained 34% of the variance in the county-level cancer death rate.

**Figure 1:**
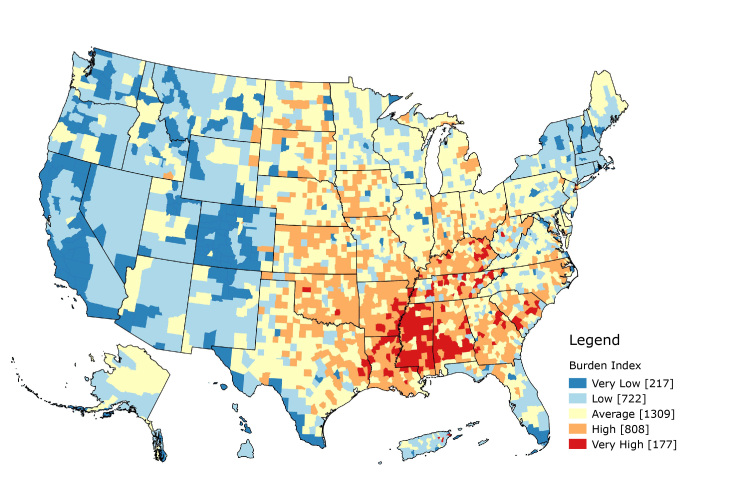
Map of burden index. Guam, American Samoa, and the North Mariana Islands are not shown.

**Geospatial distribution of the burden index and overall cancer death rate. **The burden index was created from the 9 factors described above and characterized US county and county equivalents according to their relative level of burden (Figure 1). The burden index had a mean of 0 and a standard deviation of 3.4 with values ranging from −15.33 to 15.83. Counties with the average burden fell within half of 1 standard deviation from the mean. Counties with low or high burden were more than 0.5 standard deviations from the mean but less than 1.5 standard deviations, and counties in the most extreme categories, very high and very low, were more than 1.5 standard deviations away from the mean. The index had a moderate positive association with the cancer death rate (*r* = 0.48, *P* < .001). We used the following equation to calculate burden index:

Burden index = −Factor1 + Factor2 − Factor3 + Factor4 + Factor6 + Factor7 − Factor8 + Factor10 − Factor13

In terms of population health, higher values indicated higher burden, whereas lower values indicated lower burden. Summit County, Colorado, had the lowest burden index, −15.33, whereas New York County, New York, had the highest burden index, 15.83. Slightly fewer than half of the counties fell in the average burden category (n = 1,309). More counties were classified as high or very high burden (n = 985) than low or very low burden (n = 939). Low burden counties were mostly in the Western United States, whereas high burden counties were mainly in the South and Southeast. Mississippi had the largest number of very high burden counties, with 67.1% of its counties falling in that category (n = 82), whereas Colorado had the largest number of very low burden counties, with 64.1% of its counties falling in that category (n = 64). When we combined the low with very low categories and the high with very high categories, 90.6% of Colorado’s counties occur in the low–very low category. Colorado had no counties that fell in the very high burden category. Kentucky had 76.7% of its counties in the combined high–very high category (n = 120). Texas had the largest number of average burden counties with approximately half of its counties in that category.

**Figure 2:**
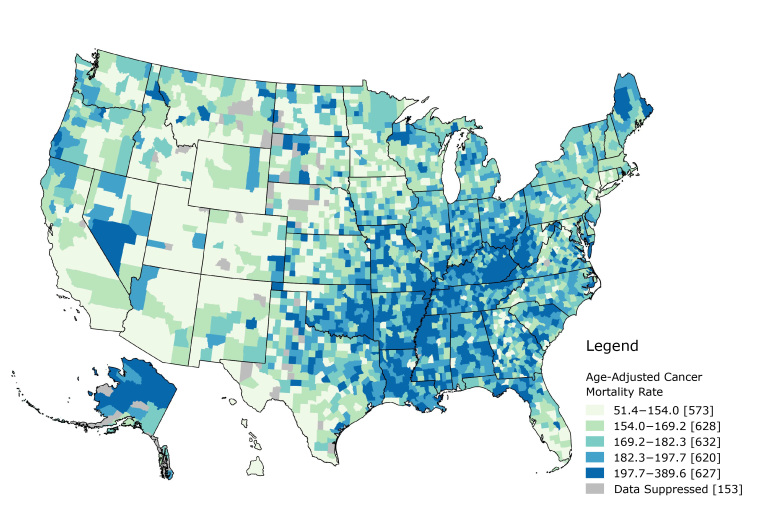
Map of overall cancer death rate using quintile breaks. Puerto Rico, the Virgin Islands, Guam, American Samoa, and the North Mariana Islands are not shown.

The overall cancer death rate was mapped by using quintile breaks (Figure 2). Rates ranged from 51.4 per 100,000 to 389.6 per 100,000. The 2 highest quintiles were concentrated in the Southeast among the following states: Missouri, Oklahoma, Arkansas, Louisiana, Mississippi, Alabama, Tennessee, Kentucky, West Virginia, and South Carolina. Alaska and Maine also had a large number of counties in the highest quintiles.

## Discussion

Our study quantifies the environmental burden of cancer via population health measures. Studies examining the burden of cancer have mainly used epidemiologic estimates (1–3). This study is the first to use the environmental burden approach to overall cancer death rates. We used data reduction to determine underlying constructs related to social determinants of health and population health indicators that contraindicate positive cancer outcomes. We developed 9 factors that were combined in an additive index. Factors are components comprising multiple variables that represent an underlying or latent construct. The burden index had a moderate association with overall cancer death rate and was limited by data availability.

Most of the variance among the counties and county equivalents was explained by demographics. The first 3 factors identified were characterized by a specific race variable as the highest loading, then additional variables. These factors alone explained 36.9% of the variance. The other variables that characterized each of the first factors showed what occurs socially in these counties. Factor 1, Asian Race, also had both medical professional density variables load on the variable, whereas factor 2, black race, also had the percentage of female-headed households, the percentage of Supplemental Nutrition Assistance Program recipients, and the poverty and unemployment rates load on it. Hospital and population density and housing were the only factors not significantly associated with overall cancer death rate.

In adjusted regression analysis, Hispanic ethnicity and isolation and housing were negative predictors. The housing factor represents poor housing outcomes. A positive value would indicate more homes that use wood for heat, have no plumbing, or more vacant homes within the county. Few studies have examined housing’s influence on cancer and focused on environmental exposures rather than these specific aspects (20). The Hispanic ethnicity and isolation factor may be a proxy for social support. Besides race and isolation, several foreign-born variables loaded positively on this factor. Other researchers have reported that despite socioeconomic disadvantages, US Hispanic residents have similar or better health outcomes than non-Hispanic white residents (21). This may only apply to foreign-born Hispanic residents, because they fare even better in terms of mortality (22). These factors might represent protective latent factors, whereas other significant factors corresponded to an increase in the overall cancer death rate, on average.

If we consider these county-level estimates as proxies for neighborhoods, we can examine how burden influences outcomes, not just mortality, along the cancer control continuum. Neighborhoods can influence general health outcomes through material deprivation, psychosocial mechanisms, health behaviors, and access to resources (23). Counties are major political and administrative units; most states use counties as their primary administrative division (24). County-level analyses provide insights that might allow for actionable changes. Mapping the burden index can demonstrate potential areas of need. A distinct geospatial pattern exists, with most high burden counties concentrated in the South and Southeast and most low burden counties concentrated in the West and Northeast. This pattern is similar to that of overall cancer mortality. Further research can follow up with additional geospatial and epidemiologic analyses. Translating the results through mapping and geographic information systems (GIS) readily conveys the relevance to public health practitioners and researchers. Both figures in our study demonstrate a clear need in the US South and Southeast, especially Alabama, Arkansas, Kentucky, Louisiana, Mississippi, Oklahoma, and Tennessee. These states have a concentration of high and very high burden counties, as well as counties that fall in the 2 highest quintiles for overall cancer mortality. Both burden and cancer mortality is concentrated in areas along the Mississippi River. Future research might examine what additional macrosocial mechanisms may cause this.

Beyond the index itself, individual factors can be parsed out and mapped, and the associations with individual factors and various cancer outcomes can be explored. Each factor provides substantially more information than a single variable would. Additional research shows that social and built environment attributes exert independent influences along the cancer control continuum (23). Kreiger (25) emphasizes the need to investigate which social determinants result in health inequities to improve understanding of etiology and grounds for action.

Although the 34% of the variance in the age-adjusted cancer death rate might seem small, cancer is a heterogeneous disease, requiring a transdisciplinary approach (26). Much literature focuses on individual-level etiology, but cancer is an enormous public health burden (23,26). Our study confirms that over a third of the variation may be associated with social environment and implies a reciprocal relationship between the individual and the environment, as the socioecological model indicates (26). Few studies evaluated and created an index for population health, and none focused on cancer burden at smaller geographic levels, such as counties, tracts, and neighborhoods. Previous studies examined social capital (27), social vulnerability to environmental hazards (9), health opportunity (28), and child opportunity (29). Future studies might quantify county-level cancer burden by using epidemiologic measures of incidence and mortality, in addition to structural equation modeling and other analytic techniques.

Our study had limitations. Principal components analysis is an unsupervised method that creates scores only for observations with complete data; imputed data are subject to the limitations of the imputation methods. This study’s results depend upon the validity of the data collected, which may lend itself to nonresponse and selection bias. In addition, some variables, such as those from the Behavioral Risk Factor Surveillance System, are model-based estimates rather than true population estimates. Finally, correlations do not imply causation, and care is needed when interpreting the index. Appropriate analyses, such as structural equation modeling or path analysis, can be conducted to infer causation rather than correlation.

Our study quantifies environmental burden through a multifactor index. These results and the variables collected for use could guide public health practice through program planning. The use of GIS facilitates visualizing and determining areas of need. The association between the index and factors and age-adjusted cancer death rate from 2010 through 2014 was explored. Results demonstrated distinct geospatial patterns of US burden and cancer mortality. These results suggest the need to examine environmental effects (social, physical, or built) on cancer mortality and suggest that this index and factors can facilitate exploration of associations with additional health outcomes. The combination of these ecological factors with individual-level information may further explain the variation in the age-adjusted cancer death rate.

## References

[1] Fitzmaurice C , Allen C , Barber RM , Barregard L , Bhutta ZA , Brenner H , ; Global Burden of Disease Cancer Collaboration. Global, regional, and national cancer incidence, mortality, years of life lost, years lived with disability, and disability-adjusted life-years for 32 cancer groups, 1990 to 2015: a systematic analysis for the global burden of disease study. JAMA Oncol 2017;3(4):524–48. 10.1001/jamaoncol.2016.5688 27918777 PMC6103527

[2] Devleesschauwer B , Havelaar AH , Maertens de Noordhout C , Haagsma JA , Praet N , Dorny P , Calculating disability-adjusted life years to quantify burden of disease. Int J Public Health 2014;59(3):565–9. 10.1007/s00038-014-0552-z 24752429

[3] Brown ML , Lipscomb J , Snyder C . The burden of illness of cancer: economic cost and quality of life. Annu Rev Public Health 2001;22(1):91–113. 10.1146/annurev.publhealth.22.1.91 11274513

[4] Thacker SB , Stroup DF , Carande-Kulis V , Marks JS , Roy K , Gerberding JL . Measuring the public’s health. Public Health Rep 2006;121(1):14–22. 10.1177/003335490612100107 16416694 PMC1497799

[5] Isfeld-Kiely H , Balakumar S . Framing burden: towards a new framework for measuring burden of disease in Canada. Winnipeg (MB): National Collaborating Centre for Infectious Diseases; 2015.

[6] National Collaborating Centre for Infectious Diseases (NCCID). More than just numbers: exploring the concept of “burden of disease.” Winnipeg (MB): National Collaborating Centre for Infectious Diseases; 2016.

[7] Parrish RG . Measuring population health outcomes. Prev Chronic Dis 2010;7(4):A71. 20550829 PMC2901569

[8] Lantz PM , Pritchard A . Socioeconomic indicators that matter for population health. Prev Chronic Dis 2010;7(4):A74. 20550832 PMC2901572

[9] Cutter SL , Boruff BJ , Shirley WL . Social vulnerability to environmental hazards. Soc Sci Q 2003;84(2):242–61. 10.1111/1540-6237.8402002

[10] Mokdad AH , Remington PL . Measuring health behaviors in populations. Prev Chronic Dis 2010;7(4):A75. 20550833 PMC2901573

[11] Peppard PE , Kindig D , Jovaag A , Dranger E , Remington PL . An initial attempt at ranking population health outcomes and determinants. WMJ 2004;103(3):52–6. 15217115

[12] Remington PL , Booske BC . Measuring the health of communities — how and why? J Public Health Manag Pract 2011;17(5):397–400. 10.1097/PHH.0b013e318222b897 21788775

[13] Bollen KA . Latent variables in psychology and the social sciences. Annu Rev Psychol 2002;53(1):605–34. 10.1146/annurev.psych.53.100901.135239 11752498

[14] Carlson BL , Cox BG , Bandeh LS . SAS macros useful in imputing missing survey data. In: SAS Institute, Inc, editors. Proceedings of the Twentieth Annual SAS Users’ Group International Conference. SAS Users Group International Conference; 1995 April 2–5; Cary (NC): SAS Institute Inc; 1995. p. 1089–94.

[15] Andridge RR , Little RJA . A review of hot deck imputation for survey non-response. Int Stat Rev 2010;78(1):40–64. 10.1111/j.1751-5823.2010.00103.x 21743766 PMC3130338

[16] National Center for Health Statistics (NCHS), Centers for Disease Control and Prevention (CDC). United States NVSS mortality data. Atlanta (GA): Centers for Disease Control and Prevention; 2014.

[17] Williams B , Onsman A , Brown T . Exploratory factor analysis: a five-step guide for novices. Australasian Journal of Paramedicine. 2014;8(3):1–13. 10.33151/ajp.8.3.93

[18] Kaiser HF . The Application of Electronic Computers to Factor Analysis. Educ Psychol Meas 1960;20(1):141–51. 10.1177/001316446002000116

[19] Gorsuch R . (1983). Factor analysis, 2nd edition. Hillsdale (NJ): Lawrence Erlbaum Associates.

[20] Van Maele-Fabry G , Gamet-Payrastre L , Lison D . Residential exposure to pesticides as risk factor for childhood and young adult brain tumors: a systematic review and meta-analysis. Environ Int 2017;106:69–90. 10.1016/j.envint.2017.05.018 28623811

[21] Ruiz JM , Steffen P , Smith TB . Hispanic mortality paradox: a systematic review and meta-analysis of the longitudinal literature. Am J Public Health 2013;103(3):e52–60. 10.2105/AJPH.2012.301103 23327278 PMC3673509

[22] Patel MI , Schupp CW , Gomez SL , Chang ET , Wakelee HA . How do social factors explain outcomes in non-small–cell lung cancer among Hispanics in California? Explaining the Hispanic paradox. J Clin Oncol 2013;31(28):3572–8. 10.1200/JCO.2012.48.6217 23960183 PMC3782149

[23] Gomez SL , Shariff-Marco S , DeRouen M , Keegan TH , Yen IH , Mujahid M , The impact of neighborhood social and built environment factors across the cancer continuum: current research, methodological considerations, and future directions. Cancer 2015;121(14):2314–30. 10.1002/cncr.29345 25847484 PMC4490083

[24] US Census Bureau. Geographic areas reference manual. Washington (DC): US Census Bureau; 1994.

[25] Krieger N . Defining and investigating social disparities in cancer: critical issues. Cancer Causes Control 2005;16(1):5–14. 10.1007/s10552-004-1251-5 15750853

[26] Hiatt RA , Breen N . The social determinants of cancer: a challenge for transdisciplinary science. Am J Prev Med 2008;35(2, Suppl):S141–50. 10.1016/j.amepre.2008.05.006 18619394 PMC10773976

[27] Rupasingha A , Goetz SJ , Freshwater D . The production of social capital in US counties. J Socio-Economics 2006;35(1):83–101. 10.1016/j.socec.2005.11.001

[28] Virginia Department of Health. Virginia health equity report 2012. Virginia Department of Health; 2012 http://www.vdh.virginia.gov/content/uploads/sites/76/2016/06/Health-Equity-Report-2012.pdf. Accessed September 24, 2018.

[29] Acevedo-Garcia D , McArdle N , Hardy EF , Crisan UI , Romano B , Norris D , The child opportunity index: improving collaboration between community development and public health. Health Aff (Millwood) 2014;33(11):1948–57. 10.1377/hlthaff.2014.0679 25367989

[30] US Department of Agriculture. Atlas of rural and small-town America. Washington (DC): Economic Research Service, US Department of Agriculture; 2012. https://www.ers.usda.gov/data-products/atlas-of-rural-and-small-town-america/. Accessed September 24, 2018.

[31] Area Health Resource Files. Washington (DC): Health Resources and Services Administration. https://aspe.hhs.gov/health-resources-and-services-administration. Accessed September 24, 2018.

[32] Mobley LR . Spatial impact factor database [dataset and code book], Atlanta (GA): Georgia State University; 2015.

[33] US Department of Agriculture. Food environment atlas. Washington (DC): Economic Research Service, US Department of Agriculture; 2011. https://www.ers.usda.gov/data-products/food-environment-atlas/. Accessed September 24, 2018.

[34] Model-based small area estimates of cancer risk factors & screening behaviors. Bethesda (MD): National Cancer Institute; 2018. http://sae.cancer.gov/nhis-brfss/methodology.html. Accessed September 24, 2018.

